# A c.89G>C p.(Gly30Ala) Variant in the *DHCR7* Gene as a Cause of a Mild Phenotype in the Smith‐Lemli‐Opitz Syndrome

**DOI:** 10.1002/mgg3.70222

**Published:** 2026-06-04

**Authors:** Júlia Martinková, Emílie Vyhnálková, Martin Schwarz, Miroslava Balaščaková, Veronika Biddle, Romana Borská, Lenka Fajkusová, Lukáš Ryba, Anna Křepelová

**Affiliations:** ^1^ Department of Biology and Medical Genetics, Second Faculty of Medicine Charles University and Motol University Hospital Prague Czech Republic; ^2^ PRENET—Laboratoře lékařské Genetiky s.r.o. Pardubice Czech Republic; ^3^ Centre of Molecular Biology and Genetics, Department of Hematology, Oncology and Internal Medicine University Hospital Brno Brno Czech Republic

**Keywords:** *DHCR7*, mild phenotype, SLOS, Smith‐Lemli‐Opitz syndrome

## Abstract

**Background:**

Smith‐Lemli‐Opitz syndrome (SLOS) is a common autosomal recessive disorder caused by pathogenic variants in the *DHCR7* gene, resulting in a deficiency of the enzyme 7‐dehydrocholesterol reductase. Two forms of SLOS are recognized based on the severity of associated symptoms. Type I is characterized by a milder phenotype, whereas type II is more severe and frequently results in fetal loss or early neonatal death. We observed two unrelated patients: a 5‐year‐old boy and a 3.5‐year‐old girl, with elevated 7‐DHC levels, a milder phenotype, and an identical genotype.

**Methods:**

Variants were confirmed by Sanger sequencing.

**Results:**

A rare heterozygous likely pathogenic variant, c.89G>C p.(Gly30Ala), and a pathogenic variant, c.964‐1G>C, in the *trans* position in the *DHCR7* gene were identified in both patients.

**Conclusion:**

The two unique patients presented here suggest that the c.89G>C p.(Gly30Ala) variant is associated with a mild SLOS phenotype, which may be overlooked and unrecognized in clinical practice. Information about this rare *DHCR7* variant is important for diagnostic awareness of SLOS, especially within the Central European population. We present clinical data and a literature review of the *DHCR7* variants associated with the mild SLOS phenotype.

## Graphical abstract

Smith‐Lemli‐Opitz syndrome (SLOS) is an autosomal recessive disorder caused by pathogenic variants in the *DHCR7* gene. Based on our observations and a review of the literature, we demonstrate that the NM_001360.2(DHCR7):c.89G>C p.(Gly30Ala) variant is associated with a mild SLOS phenotype.

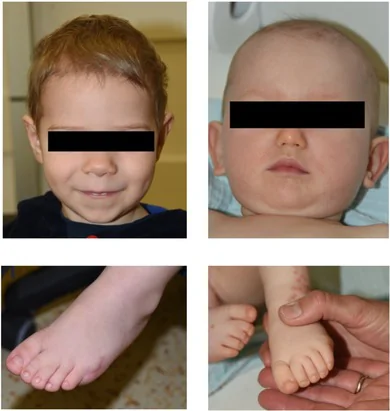

## 1 Introduction

Smith‐Lemli‐Opitz syndrome (SLOS; MIM:270400) was first described by eponymous authors in 1964 (Smith et al. 1964). It is an autosomal recessive disorder caused by a cholesterol metabolism abnormality that results from an enzyme deficiency of the 7‐dehydrocholesterol reductase (DHCR7; 3β‐hydroxysteroid‐Δ^7^‐reductase; EC 1.3.1.21; MIM:*602858). DHCR7 is encoded by the *DHCR7* gene (Nowaczyk and Wassif 1998; Fitzky et al. 1998; Moebius et al. 1998). The *DHCR7* gene is located on chromosome 11q13.2‐q13.5, spans 14 kb, and contains two 5′ non‐coding and seven coding exons (Waterham and Hennekam 2012). The *DHCR7* gene encodes a 475 amino acid polypeptide with a predicted molecular mass of 55 kDa (Moebius et al. 1998). The cDNA was cloned and sequenced, revealing that DHCR7 is a transmembrane protein comprising nine putative transmembrane segments. DHCR7 is located in the endoplasmic reticulum membrane, the site of cholesterol synthesis (Peng et al. 2018; Fitzky et al. 1998). The highest expression of the *DHCR7* mRNA was observed in the adult and fetal adrenal gland, liver, testis, and brain (Moebius et al. 1998). DHCR7 plays a crucial role in the final step of the Kandutsch‐Russell cholesterol biosynthetic pathway, the conversion of 7‐dehydrocholesterol (7‐DHC) to cholesterol (Tint et al. 1994; Irons et al. 1993; Kandutsch and Russell 1960). A diminution in DHCR7 activity has been linked to elevated levels of 7‐DHC and decreased cholesterol levels. The accumulation of the sterol precursor and the decreased essential product have been demonstrated to be toxic to the embryo (Jiang et al. 2010). Cholesterol plays an important role in the Sonic Hedgehog (SHH) signaling pathway in embryonic development of the skeletal, cardiovascular, and central nervous system (Blassberg et al. 2016). It is covalently attached to the C‐terminus of the N‐terminal domain of the HH protein during post‐translational modification, and it helps regulate tissue diffusion and the HH gradient (Xu and Tang 2022). Cholesterol also participates in the direct activation of Smoothened protein (SMO), and as a second messenger between the membrane proteins PTCH1 and SMO (Radhakrishnan et al. 2020). Conversely, cholesterol deficiency, as opposed to an accumulation of 7‐DHC, results in a dysregulation of SMO within the SHH signaling pathway (Blassberg et al. 2016).

The prevalence of SLOS has been estimated to be 1:15,000–1:60,000 within European populations (Witsch‐Baumgartner et al. 2008), with the highest incidence in the Czech Republic (1:10,000) and Slovakia (1:15,000) (Bzdúch et al. 2000). The highest carrier frequency is estimated to be approximately 2.35% (1 in 42) in Ashkenazi Jews and 1 in 54 in Northern Europeans, which classifies it as one of the most prevalent recessive disorders (Lazarin et al. 2017). It is conceivable that the incidence is more significant than previously assumed, given the existence of undiagnosed, unrecognized mild forms and fetal death in utero in the severe and lethal forms (Rojare et al. 2019; Jezela‐Stanek et al. 2008).

The phenotype is characterized by prenatal and postnatal growth restriction, microcephaly, limb malformations, postaxial polydactyly, 2–3 syndactyly of the toes, underdeveloped external genitalia in males, malformations of the affected organs, nervous system, cardiac defects, and behavioral disorders. Some patients present with severe intellectual disability, growth retardation, and failure to thrive (Nowaczyk and Irons 2012; Smith et al. 1964). Two types of SLOS have been identified: SLOS type I is distinguished by the presence of mild symptoms, while SLOS type II is more severe and frequently results in fetal demise or neonatal death (Waterham et al. 1998). A diagnosis of SLOS is made in the presence of suggestive clinical features and an elevated 7‐DHC level and/or identification of biallelic pathogenic/likely pathogenic variants in the *DHCR7* gene (Nowaczyk and Wassif 1998).

Our report details the clinical information of two mild SLOS patients carrying a rare *DHCR7* gene variant. Both patients were diagnosed postnatally at a time when SLOS was not yet considered in their differential diagnoses. Other authors discussed mild SLOS patients with the same *DHCR7* variant in the context of previous genetic analyses.

## 2 Methods

### 2.1 Editorial Policies and Ethical Considerations

The Ethics Committee of the Motol University Hospital, Prague, Czech Republic, approved the consent form. All procedures were performed according to the tenets set forth in the World Medical Association Declaration of Helsinki. Both patients underwent a molecular genetic analysis following the acquisition of written informed consent from their parents.

### 2.2 Molecular Genetic Methods

Patients' genomic DNA was isolated from peripheral blood leukocytes using a standard extraction procedure with MagCore Super (RBC Bioscience Corp., New Taipei City, Taiwan). Polymerase chain reaction (PCR) and Sanger sequencing were used to detect the variants. The analysis of the entire coding sequence of the *DHCR7* gene (exons 3–9) in Patient 1 was performed on an ABI 3130xl Genetic Analyzer (Applied Biosystems, Foster City, CA, USA) using a BigDye Terminator v3.1 Cycle Sequencing Kit (Applied Biosystems) according to the manufacturer's protocol. Electropherograms were analyzed using Chromas Lite MFC Application, version 2.6.6 (Technelysium, Australia), and Alamut Visual Plus software, version 1.6.1 (SOPHiA GENETICS, Switzerland). Patient 2 was initially examined for the diagnosis of incontinentia pigmenti using the next‐generation sequencing (NGS) panel KAPA HyperCap (Roche, USA) on the NextSeq 500 (Illumina, USA). The results were verified by Sanger sequencing in the patient and the patient's parents.

## 3 Results

### 3.1 Clinical Features—Patient 1

**FIGURE 1:**
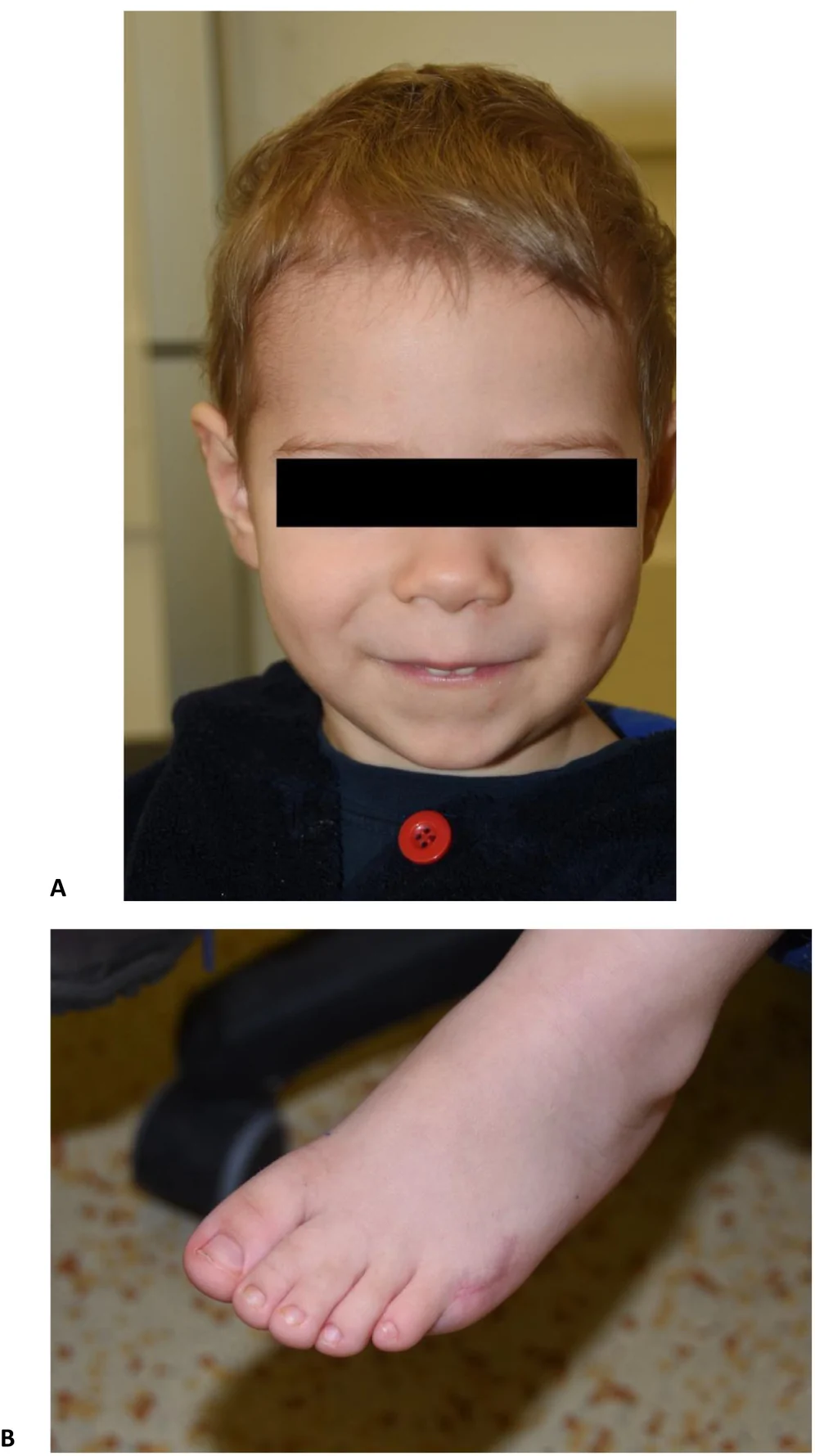
The phenotype of patient 1. (A) Mild facial dysmorphism. (B) Left foot with syndactyly and a scar after removal of the sixth toe.

The first patient is a 5‐year‐old male patient, born to non‐consanguineous parents after an uneventful pregnancy. At birth, the examination revealed the presence of postaxial hexadactyly of the left foot and hand, accompanied by bilateral syndactyly of the second and third toes. At the age of 2.5 years, the boy exhibited short stature with a height of 86 cm (7th percentile, SD −1.43), a weight of 11.6 kg (4th percentile, SD −1.43), and mild facial dysmorphism, including epicanthus, a broad nasal bridge, and borderline microcephaly (head circumference of 46.1 cm, 1st percentile, SD −2.31) (see Figure 1). He started tummy time (leaning on his forearms and lower chest) at around 3 months, rolling from back to front at around 6 months, crawling at 7 months, climbing at 9 months, and walking independently at approximately 14 months. His first word was close to 1 year old. He was very clever and managed everything in accordance with his age. Even within the framework of speech, he formed sentences and understood spoken language without any problems. No significant developmental delay or behavioral abnormalities were observed at the age of 5. Abdominal and cardiac ultrasound scans were normal. The external male genitalia were found to be without atypia. The patient's blood cholesterol level was within the normal range, with a value of 4.3 mmol/L (reference range 2.6–4.8 mmol/L) using an enzymatic colorimetric assay (CHOD‐PAP). However, the 7‐DHC level was elevated above the reference range (qualitative spectrophotometric method). The elevated 7‐DHC level, in conjunction with the presence of syndactyly, prompted the consideration of SLOS as a potential diagnosis.

### 3.2 Clinical Features—Patient 2

**FIGURE 2:**
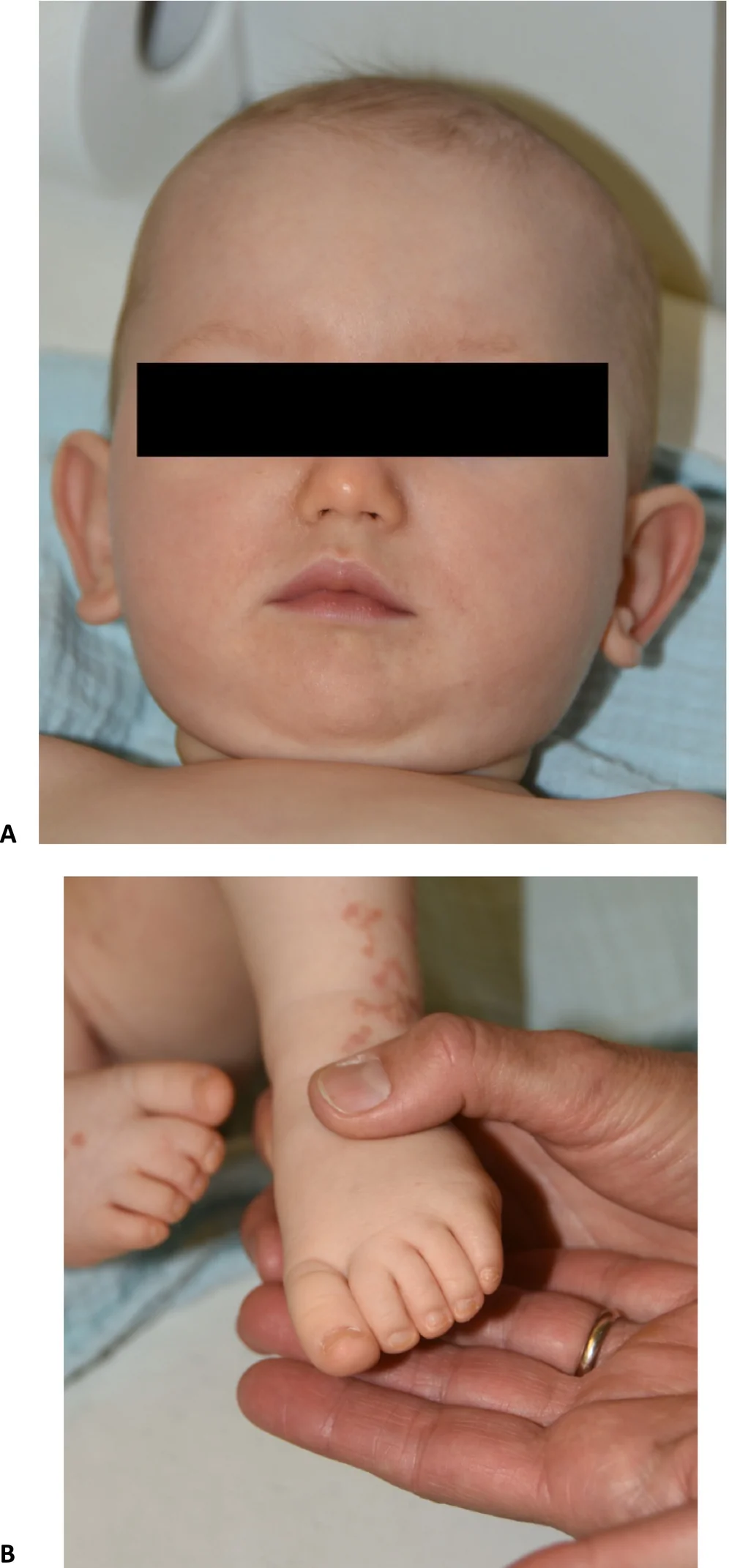
The phenotype of patient 2. (A) Mild facial dysmorphism. (B) Feet with syndactyly.

The second patient is a 3.5‐year‐old female patient with a primary diagnosis of incontinentia pigmenti (IP). The girl was born from a pregnancy of non‐consanguineous parents by elective cesarean section for borderline fetal intrauterine growth restriction (IUGR). At birth, the girl exhibited striated blisters on the limbs, corresponding to the IP diagnosis. The diagnosis was later verified at the molecular level. Additionally, she displayed syndactyly of the second and third toes on both feet (see Figure 2). At 7 months, she exhibited mild facial dysmorphism, including epicanthus, anteverted auricles, and anomalous dentition, consistent with the IP spectrum. She started back‐to‐front rolling at 7 months, crawling at 7 months, and standing up at 9 months. At the age of 9 months, her height was 69 cm (18th percentile, SD −0.92), her weight was 6.3 kg (SD −3.1), corrected to her height: 2nd percentile, SD −2.1. She exhibited microcephaly with a head circumference of 40 cm (SD −3.4, corrected to her height: SD −2.6). No other congenital anomalies were observed in the patient. At the age of 4 years, her global psychomotor development was normal, she learned new skills, and she spoke in sentences. The cholesterol level was found to be close to the lower range (2.8 mmol/L), and the 7‐DHC level was elevated. The cholesterol was measured by CHOD‐PAP, and 7‐DHC was determined by a qualitative spectrophotometric method. Consequently, cholesterol supplementation was initiated.

### 3.3 Molecular Genetic Testing

In Patient 1, two heterozygous variants, likely pathogenic NM_001360.2(DHCR7):c.89G>C p.(Gly30Ala) (rs200334114, Variation ID: 397518) in exon 3 and pathogenic NM_001360.2(DHCR7):c.964‐1G>C p.? (rs138659167, Variation ID: 93725) in intron 8 were identified in the *trans* position. The *trans* position was confirmed by segregation analysis in the patient's parents. The c.89G>C p.(Gly30Ala) variant was maternally inherited.

In Patient 2, NGS analysis revealed the presence of a *de novo* likely pathogenic variant in the *IKBKG* gene (data not shown), thus confirming the IP diagnosis. Simultaneously, the analysis identified the presence of NM_001360.2(DHCR7):c.89G>C p.(Gly30Ala) and NM_001360.2(DHCR7):c.964‐1G>C p.? variants in compound heterozygous form, as a secondary finding. The variants were verified and confirmed in the patient and her parents by targeted analysis of exons 3 and 9 using Sanger sequencing. The c.89G>C p.(Gly30Ala) variant was found to be of paternal origin.

## 4 Discussion

We observed two unrelated patients with a mild form of SLOS carrying the same *DHCR7* variants. The first detected variant, c.964‐1G>C p.?, located in intron 8, is the most prevalent pathogenic variant in the *DHCR7* gene, with an allele frequency of 30% in Caucasians with SLOS (Waterham and Hennekam 2012). The allele frequency in the European (non‐Finnish) population is 0.00935 in the Genome Aggregation Database (gnomAD) (version 4.1). The frequency of the c.964‐1G>C p.? variant in the Czech population is 1 in 167. In conjunction with the c.452G>A p.(Trp151*) variant (allele carrier frequency 1:56, in the Czech population), it constitutes 51% of all detected variants in the *DHCR7* gene in the Czech population (Witsch‐Baumgartner et al. 2008; Blahakova et al. 2007). This causal splice variant, previously described as IVS8‐1G>C, has been published several times as pathogenic. It abolishes the canonical splice site of exon 9, thereby causing aberrant splicing of the *DHCR7* mRNA at a cryptic splice acceptor site located 5′ of the mutated splice site. This results in the partial retention of a 134‐bp intron sequence and the production of a non‐functional protein (Waterham and Hennekam 2012).

The other variant, a missense c.89G>C p.(Gly30Ala) variant in exon 3, has been documented previously in two male siblings (Haas et al. 2007), in a fetus from a terminated pregnancy (Blahakova et al. 2007), in a fetus with agenesis of the corpus callosum and ventriculomegaly (Gabriel et al. 2022), in a mother with mild microcephaly and her daughter with SLOS (Perez et al. 2023), and in a female with Diamond‐Blackfan Anemia as a second condition (Tung et al. 2024) (see Table 1).

**TABLE 1:** Reported patients with c.89G>C p.(Gly30Ala) variant and a second variant in the *DHCR7* gene.

Literature	Age	Sex	Variant 1	Variant 2	Cholesterol (mmol/L)	7‐DHC (μmol/L)	Phenotype	Psychomotor development	Other conditions	Score system^c^
Patient 1—present study	5 y	M	c.89G>C p.(Gly30Ala)	c.964‐1G>C p.?	4.3	Elevated	Postaxial hexadactyly, 2–3 toe syndactyly, short stature, mild facial dysmorphism, mild microcephaly	Normal	—	2
Patient 2 ‐ present study	3.5 y	F	c.89G>C p.(Gly30Ala)	c.964‐1G>C p.?	2.8	Elevated	IUGR, 2–3 toe syndactyly, striated blisters, mild facial dysmorphism, microcephaly	Normal	IP (likely pathogenic variant in *IKBKG* gene)	1
Tung et al. (2024)	24 y	F	c.89G>C p.(Gly30Ala)	c.1087C>T p.(Arg363Cys)	—	Elevated (16.3)	—	Bipolar disorder, MCA, DD, ID	Diamond‐Blackfan Anemia (pathogenic variant in *RPL5* gene)	—
Perez et al. (2023)	33 y	F^a^	c.89G>C p.(Gly30Ala)	c.278C>T p.(Thr93Met)	—	Elevated	Mild microcephaly	Normal	—	—
Perez et al. (2023)	—	F^a^	c.89G>C p.(Gly30Ala)	c.452G>A p.(Trp151*)	—	Elevated	—	—	—	—
Gabriel et al. (2022)	Fetus	—	c.89G>C p.(Gly30Ala)	c.452G>A p.(Trp151*)	—	—	Agenesis of the corpus callosum and ventriculomegaly	—	—	—
Blahakova et al. (2007)	Fetus	—	c.89G>C p.(Gly30Ala)	c.452G>A p.(Trp151*)	—	Elevated	—	—	—	—
Haas et al. (2007)	—	M^b^	c.89G>C p.(Gly30Ala)	c.964‐1G>C p.?	4	Elevated (51.7)	—	—	—	5
Haas et al. (2007)	—	M^b^	c.89G>C p.(Gly30Ala)	c.964‐1G>C p.?	4.5	Elevated (118.9)	—	—	Sleep disturbance under simvastatin treatment	5

*Note:* Ref. Sequence GenBank NM_001360.2.

Abbreviations: 7‐DHC, 7‐dehydrocholesterol reductase; DD, developmental delay; F, female; ID, intellectual disability; IP, incontinentia pigmenti; IUGR, intrauterine growth restriction; M, male; MCA, multiple congenital anomalies; SLOS, Smith‐Lemli‐Opitz syndrome; y, years.

^a^ Mother and daughter.

^b^ Siblings.

^c^ According to score system < 20 for mild phenotype of SLOS by Kelley and Hennekam (2000).

Haas et al. (2007) first reported the same compound heterozygous variants, c.964‐1G>C p.? and c.89G>C p.(Gly30Ala), in two male siblings. Utilizing the Kelley and Hennekam scoring system (Kelley and Hennekam 2000), the variant was categorized as mild (score 5), with no accompanying description of the phenotypic characteristics exhibited by the siblings. Our patients scored 2 points (boy) and 1 point (girl), respectively, consistent with the mild SLOS phenotype according to the Kelley and Hennekam scoring system, and 10 points (boy) and 5 points (girl) according to the universal severity score system by Nowaczyk and Irons (2012).

In the same year, Blahakova et al. (2007) described a fetus with the c.89G>C p.(Gly30Ala) and c.452G>A p.(Trp151*) variants and found elevated levels of 7‐DHC in the amniotic fluid. The pregnancy was terminated due to an increased risk of SLOS. However, the publication does not describe the phenotype of the fetus.

In a separate case, Perez et al. (2023) reported a 33‐year‐old female with normal psychomotor development and no major or minor anomalies except for mild microcephaly. The c.89G>C p.(Gly30Ala) and c.278C>T p.(Thr93Met) variants were detected. Furthermore, elevated plasma concentrations of 7‐ and 8‐DHC were observed, confirming the SLOS diagnosis. The patient's daughter carried a maternally inherited c.89G>C p.(Gly30Ala) variant and a paternally inherited c.452G>A p.(Trp151*) variant. She was clinically diagnosed with SLOS. Reverse phenotyping was employed in the patient, and the observation by the authors supports the statement that the c.89G>C p.(Gly30Ala) variant, in conjunction with the second compound c.278C>T p.(Thr93Met) variant, is associated with a mild form of SLOS in the patient. Conversely, the c.89G>C p.(Gly30Ala) variant, when combined with the c.452G>A p.(Trp151*) variant, was found to be associated with SLOS in the patient's daughter. We found c.89G>C p.(Gly30Ala) and c.964‐1G>C p.? variants in our patients. In contrast, Blahakova et al. (2007) reported a second compound c.452G>A p.(Trp151*) variant, and Perez et al. (2023) reported c.278C>T p.(Thr93Met) and c.452G>A p.(Trp151*) variants. The combinations above have the potential to result in the manifestation of diverse phenotypes (Bialer et al. 1987). The homozygous form of the c.89G>C p.(Gly30Ala) variant has not yet been published; therefore, it is hypothesized that it may result in a very mild SLOS phenotype or potentially remains asymptomatic. Waterham and Hennekam (2012) posited that approximately 63% of patients diagnosed with SLOS have been described as having a combination of a missense and a nonsense variant, while 30% have been found to have two missense variants. The remaining 6% consists of two nonsense variants.

Several other publications reported the c.89G>C p.(Gly30Ala) variant, yet detailed clinical information is lacking. Gabriel et al. (2022) described a fetus with agenesis of the corpus callosum and ventriculomegaly that carried the c.452G>A p.(Trp151*) variant as a second one (see Table 1). Additionally, the publication by Waterham and Hennekam (2012) on the SLOS mutation spectrum refers to the publication by Blahakova et al. (2007). The reverse phenotyping was also used in a 24‐year‐old female patient who carried the c.89G>C p.(Gly30Ala) and c.1087C>T p.(Arg363Cys) variants in the *DHCR7* gene. This female patient was diagnosed with the clinical features of both SLOS and Diamond‐Blackfan anemia (Tung et al. 2024).

There are a few documented cases in which the c.89G>C p.(Gly30Ala) variant was identified as a standalone variant, with the patient being a carrier only. Faldynová et al. (2023) identified the variant in a pregnant woman through non‐invasive prenatal testing. Another report was by Cross et al. (2015) using massively parallel sequencing datasets. The heterozygous form of the c.89G>C p.(Gly30Ala) variant has been referenced in a patient with complete androgen insensitivity syndrome (Kolesinska et al. 2018) and a patient with autism (Saskin et al. 2017). In both cases, the authors classified the variant as having uncertain significance. Concurrently, Mirabello et al. (2020) and Kim et al. (2021) reported this likely pathogenic variant in patients diagnosed with osteosarcoma and leukemia. Variant was also detected in retrospective analysis in the Czech and Hungarian cohort using NGS, reported by Kovács et al. (2026).

The mild phenotype of SLOS has also been documented in conjunction with specific other variants in the *DHCR7* gene (Supporting Information).

Currently, there is limited information regarding the functional impact of the c.89G>C p.(Gly30Ala) variant, as no functional studies have been performed to date. We propose the following hypothesis to explain the mild clinical presentation associated with this variant. A unique feature of glycine is the absence of a side chain, which allows a broader range of torsion angles compared to other amino acids, including alanine. This enhances the conformational flexibility of the amino acid backbone, making glycine particularly prevalent and useful in tight loops, turns, or flexible linkers between domains (Yan and Sun 1997). Based on an analysis of the AlphaFold2 (Jumper et al. 2021) model of DHCR7 (AlphaFold Protein Structure Database Code: Q9UBM7) and the structural homolog Delta(14)‐sterol reductase from *Methylotuvimicrobium alcaliphilum* (PBD codes: 4QUV) (Li et al. 2015), it seems plausible that Gly30 flexibility may be essential for both proper protein folding and protein activity. Its flexibility potentially allows the incorporation of the side chain of the preceding amino acid (Trp29) into a hydrophobic pocket, facilitating the transition from the disordered N‐terminus of the protein to the folded transmembrane domain. Furthermore, due to its position at the edge of the active site entry, it may play a role in gating the protein's active site (Yan and Sun 1997; Li et al. 2015). In conclusion, while the p.Gly30Ala variant is predicted to lead to suboptimal protein folding and reduced functional enzyme levels, these structural insights remain hypothesis‐generating and require further experimental validation through functional studies, fibroblast sterol profiling or enzymatic activity assays. These alterations are likely to exert an influence on the SHH signaling pathway, albeit with a mild effect. The cholesterol levels in our patients were found to be within the normal range, a factor that likely contributed to the milder clinical phenotype, thereby resulting in the absence of severe birth defects or brain anomalies. As Blassberg et al. (2016) have previously described, cholesterol deficiency resulting from the loss of DHCR7 enzymatic activity has an impact on SHH signaling pathway dysregulation. Further investigation is needed to determine the variant's impact within SHH pathways.

The *in silico* predictions using bioinformatics tools integrated into VarSome Premium software (https://varsome.com/) assigned the variant the following rankings: pathogenic (12), variant of uncertain significance (VUS) (16), and benign (3) (on‐demand programs). The ClinVar database assigned: pathogenic (3), likely pathogenic (8), and VUS (2). The Leiden Open Variation Database (LOVD) (https://databases.lovd.nl/shared/genes/DHCR7) classified it as likely pathogenic, and the Human Gene Mutation Database (HGMD) (http://www.hgmd.cf.ac.uk) as a disease‐causing mutation. The allele frequency in the European (non‐Finnish) population is 0.000166 (gnomAD, version 4.1). Based on the available evidence, we have evaluated the c.89G>C p.(Gly30Ala) variant as likely pathogenic variant (*class 4*) according to the American College of Medical Genetics and Genomics (ACMG) and the Association for Molecular Pathology (AMP) guidelines (PM5, PM2, PP3, and PP4) (Richards et al. 2015), causally implicated in the milder form of SLOS (all assessment results were updated as of March 2026).

Two additional variants have been described at the Gly30 codon site: c.88G>A p.(Gly30Ser) (Variation ID: 1934663) and c.89G>A p.(Gly30Asp) (Variation ID: 2720743). These have been classified as likely pathogenic. Neither of these variants has been reported previously in the literature in SLOS‐affected patients. Advanced modeling of protein sequence and biophysical properties, performed at Labcorp Genetics (formerly Invitae), indicates that these missense variants are expected to disrupt the function of the DHCR7 protein. The alterations in amino acid character are as follows: a neutral and non‐polar amino acid is replaced by serine, which is neutral and polar, or an acidic and polar amino acid, aspartic acid, is introduced (available at https://clinvarminer.genetics.utah.edu).

One of the most frequently observed clinical manifestations of a mild form of SLOS is the presence of syndactyly of the second and third toes, as well as the manifestation of subtle dysmorphic features. Syndactyly is present in more than 95% of patients diagnosed with SLOS. However, it is not exclusive to SLOS, and its connection with SLOS may thus be overlooked. The elevated levels of 7‐DHC are regarded as the primary indication for molecular genetic testing (Jezela‐Stanek et al. 2008). Detection of elevated levels of 7‐DHC using a quantitative spectrophotometric analysis is a commonly used method in patients with suspected SLOS. However, it should be noted that while this method is highly specific and correlates strongly with mass spectrometry (Scalco et al. 2003), it does not provide the precise quantification of 7‐DHC and 8‐DHC levels required for detailed sterol ratios. Furthermore, the clinical utility of precise sterol quantification may be diminished in older patients. Given the ages of our patients (3.5 and 5 years), their sterol profiles may have undergone physiological shifts that differ from established diagnostic baselines typically observed in neonates. Consequently, we advocate for submitting specimens to specialized laboratories for precise concentration analysis at the time of initial diagnosis. According to the modified Kelley and Hennekam scoring system (Kelley and Hennekam 2000), patients who score low are classified as mild (i.e., severity score of less than 20). Nevertheless, it appears that this scoring system is not reliable for SLOS type I. The scoring system is designed to assign substantial weight to physical malformations, as Jezela‐Stanek et al. (2008) noted. This limits the ability to identify cases with less severe malformations, and it does not consider the severity of intellectual disability, behavioral and sleep disturbances, or feeding difficulties (Nowaczyk and Irons 2012). The cases presented here deviate from the typical presentation of the syndrome in that the patients exhibited an absence of organ defects and demonstrated a typical psychomotor developmental trajectory at 3.5–5 years of age. Tint et al. (1995) suggested that the decisive factor in the development of SLO types I and II is apparently the presence of specific *DHCR7* variants. These patients illustrate the phenotypic variability inherent in genetic disorders, thereby complicating our understanding of SLOS.

## 5 Conclusion

We describe two patients with a mild SLOS phenotype carrying the c.964‐1G>C p.? and c.89G>C p.(Gly30Ala) variants in the *DHCR7* gene. This clinical observation expands the knowledge base regarding the c.89G>C p.(Gly30Ala) variant. Our findings suggest that this rare, likely pathogenic variant is potentially associated with a milder form of SLOS. These results may assist clinical geneticists in achieving more accurate diagnostic outcomes. While the identification of such rare variants is valuable for improving diagnostic yields, particularly within the Central European population, where this variant has now been documented in our two patients, Blahakova et al. (2007), Faldynová et al. (2023), and Kovács et al. (2026), broader implications for population‐wide screening require further study. A milder form of SLOS is frequently overlooked in clinical practice. Our two patients presented with a mild SLOS phenotype and a low severity score, and did not show signs of delayed early psychomotor development or organ defects at the time of diagnosis. However, as the patients are in early developmental stages (ages 3.5 and 5 years), more subtle developmental and behavioral issues may become more perceptible with age. Furthermore, it should be noted that a 100% correlation between genotype and phenotype is not applicable to SLOS, and clinical outcomes may vary significantly even with identical genotypes. This implies that some patients exhibiting low scores may still present with significant developmental delay and behavioral problems. We consequently support the recommendation proposed by Jezela‐Stanek et al. (2008) that all patients exhibiting significant toe syndactyly and subtle facial dysmorphic features specific to SLOS should undergo biochemical testing for 7‐DHC and molecular genetic testing of the entire *DHCR7* gene, even in the absence of strong clinical suspicion of SLOS. Early diagnosis of SLOS offers significant therapeutic benefits through cholesterol supplementation therapy.

## Author Contributions

Júlia Martinková was responsible for the content, formal analysis, writing, and editing of the manuscript, data analysis and interpretation in Patient 1, and conducted the literature review. Emílie Vyhnálková, Lukáš Ryba, and Martin Schwarz conducted the genetic consultations and wrote the clinical part of the manuscript. Veronika Biddle revised the manuscript and helped critically evaluate the biochemical impact of the variant. Lenka Fajkusová and Romana Borská performed NGS and Sanger sequencing and interpreted the results for Patient 2. Miroslava Balaščaková and Anna Křepelová edited and revised the manuscript. Anna Křepelová approved the final version of the manuscript. All authors agreed to take personal responsibility for their contributions. All authors read and approved the final version of the manuscript.

## Funding

This article was supported by grants from the Czech Ministry of Health: MH CZ—DRO, Motol University Hospital, Prague, Czech Republic 00064203; and MH CZ—conceptual development of research organization (FNBr, 65269705).

## Ethics Statement

This article was approved by the Ethics Committee of Motol University Hospital, Prague, Czech Republic.

## Consent

The parents of our patients were provided with written information, and their consent to participate in the study was obtained. The parents also provided informed consent for the publication of the photographs.

## Conflicts of Interest

The authors declare no conflicts of interest.

## Supporting information


**Data S1:** The comprehensive table details previously documented *DHCR7* variants associated with mild SLOS phenotypes, including clinical presentations and corresponding hormone profiles.

## Data Availability

The data that support the findings of this study are available from the corresponding author upon reasonable request.
